# Hepatitis E Virus in Pigs from Slaughterhouses, United States, 2017–2019

**DOI:** 10.3201/eid2602.191348

**Published:** 2020-02

**Authors:** Harini Sooryanarain, Connie L. Heffron, Dolores E. Hill, Jorrell Fredericks, Benjamin M. Rosenthal, Stephen R. Werre, Tanja Opriessnig, Xiang-Jin Meng

**Affiliations:** Virginia Polytechnic Institute and State University, Blacksburg, Virginia, USA (H. Sooryanarain, C.L. Heffron, S.R. Werre, X.-J. Meng);; US Department of Agriculture, Beltsville, Maryland, USA (D.E. Hill, J. Fredericks, B.M. Rosenthal);; University of Edinburgh, Midlothian, Scotland, UK (T. Opriessnig)

**Keywords:** Hepatitis E virus, HEV, zoonoses, genotype, subgenotype, HEV IgG, pork safety, market-weight pigs, slaughterhouse, United States

## Abstract

Hepatitis E virus (HEV) RNA was detected in 6.3% and HEV IgG in 40% of 5,033 serum samples from market-weight pigs at 25 slaughterhouses in 10 US states. The prevalent HEV genotype was zoonotic genotype 3, group 2. Blood of HEV-viremic pigs from slaughterhouses may contaminate pork supply chains.

Hepatitis E virus (HEV; family *Hepeviridae*, genus *Orthohepevirus* *A*) comprises at least 8 distinct genotypes (*1*). In industrialized countries, swine HEV of the zoonotic genotypes 3 and 4 (HEV-3 and HEV-4) is an emerging foodborne pathogen, transmitted by consumption of raw or undercooked pork (*2*). Recently, HEV-3 has been detected in human blood donors in the United States (*3**,**4*). We previously showed that HEV-3 is present in US swine herds (*5*) and that a small proportion of commercial pork products, such as liver and chitterlings, from US grocery stores contain infectious HEV (*6*). However, the current HEV infection status of US market-weight pigs at the time of slaughter, the entry point to the food supply chain, remains unknown. We therefore investigated the presence of HEV RNA and HEV IgG prevalence in 5,033 serum samples from market-weight pigs at 25 slaughterhouses in 10 US states.

## The Study

During 2017–2019, a comprehensive set of archived serum samples from 22,940 market-weight pigs from 25 slaughterhouses in 10 US states was collected for an unrelated prevalence study of *Toxoplasma* and *Trichinella*. The samples were collected from slaughterhouses processing adult market-weight pigs >6 months of age: ≈250-pound market-weight hogs 6 months of age and female pigs >1 year of age. The blood samples were collected on the kill floor at the slaughterhouses, and serum was separated and stored frozen (−20°C) at the US Department of Agriculture–Agricultural Research Service, Beltsville Agricultural Research Center (Beltsville, MD, USA). 

For our study, an aliquot of frozen serum samples was sent to Virginia Polytechnic Institute and State University (Blacksburg, VA, USA). From a total of 22,940 samples available, we performed a stratified random selection of 5,033 samples for this study, using the SURVEYSELECT procedure in SAS version 9.4 (https://www.sas.com); a combination of state and ZIP code of origin constituted the strata. To detect HEV RNA, we used an established quantitative reverse-transcription PCR (qRT-PCR) (*7*) and a nested RT-PCR (*5*). To detect HEV IgG, we used a commercial PrioCHECK Porcine HEV Ab ELISA kit (https://www.thermofisher.com), according to the manufacturer’s protocol.

Results of qRT-PCR indicated that ≈6.3% (318/5,033; 95% CI 5.6%–7.0%) of the market-weight pigs from US slaughterhouses were viremic for HEV RNA at the time of slaughter (Table). Viral loads ranged from <100 to 10^6^ copies/mL (mean 8,285 copies/mL; 95% CI 6,210.7–25,397.2 copies/mL). The percentage of HEV-viremic pigs varied among slaughterhouses (range 0%–17.4%) and among states (Table). Higher serum HEV RNA positivity was found in pigs from 3 slaughterhouses in Iowa (17.4%, 9.5%, and 8.3%), 2 in Illinois (8.5% and 7.5%), 1 in North Carolina (7.9%), and 1 in Pennsylvania (7.5%).

**Table:** Detection of HEV IgG and RNA in serum of market-weight pigs from 25 slaughterhouses in 10 US states, 2017–2019*

State, slaughterhouse no.	No. samples tested	Positive for HEV IgG by ELISA, no. (%)	Positive for HEV RNA by qRT-PCR, no. (%)	HEV RNA, copies/mL, range
Oklahoma	455	269 (59.1)	24 (5.3)	<100–1 × 10^4^
Tennessee	56	32 (57.1)	1 (1.8)	<100
Virginia	213	89 (41.8)	6 (2.8)	<100–4 × 10^5^
Illinois				
1	40	18 (45)	3 (7.5)	102–3,083
2	55	22 (40)	3 (5.5)	<100
3	40	16 (40)	0	0
4	445	118 (26.5)	38 (8.5)	<100–3 × 10^5^
5	259	60 (23.3)	2 (0.8)	100
Wisconsin	20	9 (45)	0	0
Iowa				
1	379	135 (35.6)	36 (9.5)	<100–1 × 10^4^
2	455	304 (66.8)	23 (5.1)	<100–3 × 10^4^
3	70	21 (30)	1 (1.4)	10^3^
4	105	39 (37.1)	1 (1)	<100
5	180	67 (37.2)	15 (8.3)	<100–1 × 10^5^
6	37	17 (46.0)	0	0
7	453	153 (33.8)	79 (17.4)	<100–2 × 10^5^
8	22	5 (22.7)	0	0
Minnesota	233	76 (32.6)	10 (4.3)	<100–1 × 10^4^
North Carolina				
1	245	61 (24.9)	7 (2.9)	<100–3 × 10^4^
2	482	266 (55.2)	38 (7.9)	<100–1 × 10^5^
Nebraska				
1	223	62 (27.8)	2 (0.9)	100 – 300
2	241	66 (27.49)	9 (3.8)	<100–1 × 10^5^
Pennsylvania				
1	50	0	1 (2)	200
2	35	8 (22.9)	1 (2.9)	<100
3	240	94 (39.2)	18 (7.5)	<100–1 × 10^6^
Total	5,033	2,007 (39.9)	318 (6.3)	<100–1 × 10^6^

*HEV, hepatitis E virus; qRT-PCR, quantitative reverse-transcription PCR.

To determine HEV genotype in US slaughterhouse pigs, we further tested the 318 serum samples positive by qRT-PCR by using an established nested RT-PCR (*5*). We successfully amplified the HEV capsid gene region (348 bp) from 182 samples. Subsequent sequencing and phylogenetic analyses revealed that all 182 HEV sequences belonged to the zoonotic HEV-3 genotype and clustered within group 2 (HEV-3abchij) (Figure). The HEV sequences from slaughterhouse pigs shared ≈90%–94% nt sequence identity with the previously reported US HEV-3 isolates (GenBank accession nos. JN837481 and KF719308). We did not detect any HEV-3-efg subgenotype or HEV-4.

**Figure:**
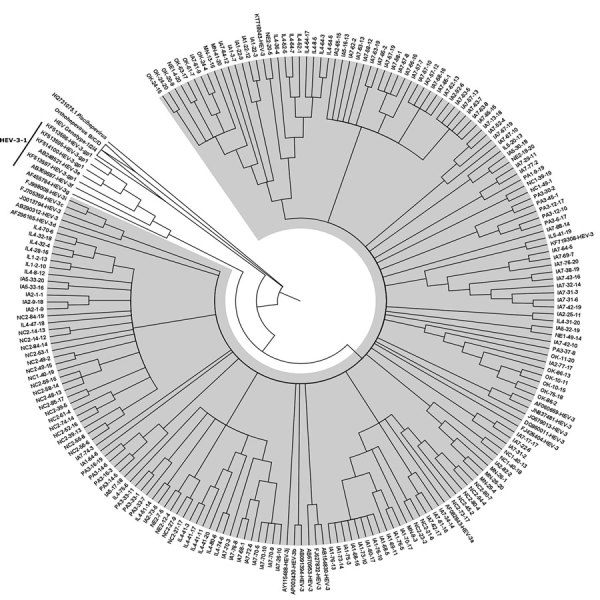
Phylogenic tree of the capsid gene region of reference HEV-1, HEV-2, HEV-3, and HEV-4 strains within species *Orthohepevirus A*, representative HEV strains from species *Orthohepeviruses B*, *C*, and *D*, as well as the cutthroat trout virus in the genus *Piscihepevirus*. The phylogenetic analysis was performed by using MEGA6 software (http://www.megasoftware.net) and the maximum-likelihood bootstrap method based on the Tamura-Nei model (*1*). The figure represents a cladogram. The HEV-3abchij sequences belonging to HEV-3 group 2 (HEV-3–2) are highlighted in gray (n = 182 sequences from slaughterhouse pigs in this study; n = 19 reference sequences from the GenBank database). The HEV-3efg sequences belonging to HEV-3 group 1 (HEV-3–1) are shown on a white background (n = 7 reference sequences from the GenBank database). Reference HEV sequences from genotypes 1/2/4 clade of *Orthohepevirus* *A*, and *Orthohepevirus* *B*/*C*/*D* clade are also shown as collapsed branches, and the cutthroat trout virus within the genus *Piscihepevirus* is shown as a separate clade. The HEV-3 reference sequences used in the phylogenetic analysis include HEV-3a AF082843, HEV-3b AP003430, HEV-3c FJ705359, HEV-3d AF296165–7, HEV-3e AB248521, HEV-3f AB369687, HEV-3g AF455784, HEV-3h JQ013794, HEV-3i FJ998008, HEV-3j AY115488, HEV-3 AB290312. We also included the following other HEV-3 sequences in the phylogenetic analysis, which were some of the top candidates from our initial BLAST (https://blast.ncbi.nlm.nih.gov/Blast.cgi) analysis: AB091394-HEV-3, AB154830-HEV-3, AB670953-HEV-3, DQ860011-HEV-3, FJ426404-HEV-3, FJ527832-HEV-3, JN837481-HEV-3, KF719308-HEV-3, KT718043-HEV-3. HEV, hepatitis E virus.

We found that the national average of HEV seropositivity among market-weight pigs in US slaughterhouses is ≈40% (95% CI 38.5%–41.2%). Seroprevalence varied from slaughterhouse to slaughterhouse and from state to state (range 0%–66.8%) (Table). At 1 slaughterhouse in Pennsylvania, all 50 pigs tested were seronegative. Higher HEV seropositivity was found at slaughterhouses in Iowa (68.8%), Oklahoma (59.1%), Tennessee (57.1%), and North Carolina (55.2%) (Table).

HEV RNA positivity (17.4%) and seropositivity (68.8%) were highest at 1 slaughterhouse in Iowa. Of note, HEV seropositivity was higher in serum samples from Tennessee, but only 1.8% of these samples were positive for HEV RNA (Table). We performed the Spearman correlation by using SAS version 9.4 and found no apparent correlation between HEV antibody seropositivity and serum HEV RNA positivity in this study (Spearman correlation R^2^ = 0.07); among 2,007 HEV IgG–positive samples, only 145 were also positive for HEV RNA (7.2%, 95% CI 6.1%–8.3%). 

## Conclusions

HEV-3 and HEV-4 are zoonotic viruses that infect pigs and humans. In this study, we found that ≈40% of US slaughterhouse pigs were seropositive for HEV, indicating prior HEV infection of the pigs on the farms, which was consistent with prior estimates for farmed US pigs (*8**,**9*). Despite the relatively high seropositivity, however, only a small proportion (6%) of the pigs had detectable HEV viremia, probably because HEV viremia is transient and thus the window for detecting HEV RNA in serum is narrow. In addition, active HEV infection occurs naturally in most farm pigs around 2 months of age (*5**,**9*). Therefore, most market-weight pigs >6 months of age at the time of slaughter are no longer actively infected by HEV. Nevertheless, studies have shown that 5.7% of UK (*10*), and 44.4% of Scotland (*11*) slaughterhouse market-weight pigs were viremic. A growing number of reported cases of autochthonous HEV-3 and HEV-4 human infection have been attributed to consumption of raw or undercooked pork (*12**,**13*), including 1 case acquired from imported HEV-4 in the United States (*14*).

The HEV sequences we detected all belonged to the zoonotic HEV-3 group 2 (HEV-3abchij). This finding is consistent with previous reports of detection of HEV-3 in US farm pigs and commercial pork products (*5**,**6*). Pigs in Europe are reportedly infected with HEV-3c, 3e, 3f, 3h, and 3i. Because our phylogenetic analysis was based on partial sequences, our results identified the prevalent HEV genotype at the group level, but more detailed study based on full-length sequence is warranted to definitively identify viral heterogeneity as well as the molecular clock of HEV evolution across the United States. Cases of autochthonous human infection with HEV-3abchij have been reported in the United Kingdom (*13*), and low levels of HEV-3abchij RNA were detected in human blood donor plasma samples in the United States (*4*). That ≈6% of slaughterhouse pigs are still viremic for HEV-3abchij raises a potential concern about pork safety because blood containing infectious HEV during slaughter may contaminate raw pork products. Therefore, to prevent foodborne HEV infection, pork should be properly cooked before consumption; an internal temperature of 71°C inactivates infectious HEV (*15*).
